# Study of Relationship Between Depression and Quality of Life in Patients With Chronic Schizophrenia

**DOI:** 10.5539/gjhs.v8n3p224

**Published:** 2015-08-06

**Authors:** Najmeh Abedi Shargh, Bahareh Rostami, Bahareh Kosari, Zakiye Toosi, Ghazaleh Ashrafzadeh Majelan

**Affiliations:** 1 Health Promotion Research Center, Zahedan University of Medical Sciences, Zahedan, Iran; 2 Department of Psychology, Neyshabur Branch, Islamic Azad University, Neyshabur, Iran; 3 Department of Clinical Psychology, Shahrood Branch, Islamic Azad University, Shahrood, Iran; 4 Zahedan University of Medical Sciences, Zahedan, Iran; 5 Department of Clinical Psychology, Zahedan Branch, Islamic Azad University, Zahedan, Iran

**Keywords:** depression, Quality of life, chronic schizophrenia

## Abstract

Depression is among the personality traits of schizophrenic patients, which results from psychotic features or is a consequence of a period of psychosis. Depression in schizophrenic patients is one of the important factors affecting their quality of life. The study population of this descriptive and analytic study consists of patients with chronic schizophrenia in Zahedan in 2014. The sample included 60 patients who simultaneously suffered from depression and were selected using random sampling (30 males and 30 females). The research instruments included the Schizophrenia Quality of Life Scale (SQLS) and the Beck Depression Inventory (the inventory was filled out by the tester). In order to form a statistics analysis, we used Pearson correlation and regression multivariate. Investigating the study hypotheses showed that there was a negative correlation between the high level of depression and low quality of life. the relationship between depression and the quality of life subscales showed that in women, the variable of symptoms and complications was a significant predictor; however, the other two variables (energy and motivation and psychosocial) were not significant predictors. In case of men, psychosocial variable was a significant predictor; however, the other two variables (energy and motivation and symptoms and complications) were not significant predictors. In general, depression on these patients makes discontent of life on them; therefore, elimination of their depression on their treatment is necessary.

## 1. Introduction

Depression in schizophrenic patients is one of the important factors affecting their quality of life (Dan et al., 2011). In general, depression in schizophrenic patients leads to dissatisfaction with the quality of life. Therefore, depression in such patients should be treated. Research shows that efforts to reduce the symptoms of depression in schizophrenia may improve the quality of life in such patients (Cotton et al., 2010; Bow-Thomas, 1999). In their study, Cotton et al., found that depression was the strongest predictor of quality of life. Symptoms of depression in schizophrenia are common and they can occur at any stage of this disorder. Moreover, drug therapy in schizophrenic patients must be accompanied by psychosocial interventions. Early diagnosis and timely interventions improve the quality of life and reduce the severity of the illness in such patients (Babinkostova et al., 2011). The close relationship between major depression and schizophrenia supports the hypothesis that these two disorders may be from the same pathology or be a result of it (Hafner, 2005; Muller et al., 2006). Symptoms of depression are among the major risk factors for suicide in such patients (Yan, 2012; Kao et al., 2011). According to Kao et al. (2011), schizophrenia is associated with high risk of suicide, depression, and psychopathic symptoms; and low quality of life–especially dissatisfaction with social relations–must be considered while assessing the suicide risk in such patients. Therefore, depression in such patients should be treated. Given the frequency of schizophrenic patients and the high cost of treating them at health and psychiatric centers, providing facilities for patients to recover and adapt themselves to their condition (disease acceptance and tolerance) and improving their quality of life is of great importance. In addition to severe disease symptoms, schizophrenic patients, who are hospitalized and treated at psychiatric centers for many years, also suffer from the pain of being away from their families, acquaintances, and relatives. In fact, they will suffer from social isolation and feeling of frustration after a while and they will eventually become depressed and indifferent, which reduces their quality of life (Fadayi, 2004). Given the aforesaid factors, this study aims to investigate the relationship between depression and quality of life in patients with chronic schizophrenia.

## 2. Method

The study population of this descriptive and analytic study consists of patients with chronic schizophrenia in Zahedan in 2014. Based on the conducted studies (14), the sample included 60 patients who simultaneously suffered from depression and were selected using random sampling (30 males and 30 females). The inclusion criteria were as follows: being diagnosed with schizophrenia for at least a year, being ate the age 30-60, willingness for participation in the study, and being able to communicate with the researcher appropriately. The research instruments included the Schizophrenia Quality of Life Scale (SQLS) and the Beck Depression Inventory (the inventory was filled out by the tester). The SQLS consisted of 30 questions assessing the quality of life in schizophrenic patients in three areas, including psychosocial (15 questions), energy and motivation (7 questions), and symptoms and complications (8 questions). Answer choices included never (0 point), rarely (1 point), sometimes (2 points), often (3 points), and always (4 points). Therefore, those gaining more points possess a lower quality of life, and adversely, fewer points indicate a higher quality of life. Research has proven this questionnaire to be of high reliability and validity for assessment of the quality of life in schizophrenic patients. This questionnaire has been standardized for Iranian cultural context. Content validity check was conducted in order to validate the questionnaire (by asking 10 experts to provide their opinion), test-retest was used to assess its reliability, and its reliability (r=0.89) was confirmed (Fruzande, 1999).

The Beck Depression Inventory (BDI-II) consisted of 21 questions, which was designed to assess feedbacks and symptoms of depression in patients. This scale determines different degrees of depression from mild to severe. The scores of this inventory range from 0 to 63. Beck et al., (1996) obtained the 1-week test-retest reliability coefficient as 0.93. In Iran, studies conducted by Partovi (1976), Vahabzadeh (1974), and Chegini (2003) showed that the reliability of the Beck Depression Inventory was high, ranging from 0.70 to 0.90. The following scores can be applied to show the overall level of depression: 0-13 (no or minimal depression), 14-19 (mild depression), 20-28 (moderate depression), and 29-63 (severe depression) (Azkhosh, 2008).

## 3. Results

In this section, demographic characteristics are described using the frequency and percentage indices. The subjects’ demographic characteristics are shown in table 1-4. The study group consisted of 30 women (50%) and 30 men (50%). In terms of age, the group consisted of seventeen 20-30 year-old individuals (28.3%), forty 40-50 year-old individuals (50%), and thirteen 50-60 year-old individuals (21.7%). In terms of marital status, 42 (70%) and 18 (30%) were single and married respectively. In terms of length of stay in the center, 1 (1.7%), 14 (23.3%), 24 (40%), 11 (18.3%), 8 (13.3%), and 2 (3.3%) stayed there for less than 2 years, 2-4 years, 4-6 years, 6-8 years, 8-10 years, and more than 10 years respectively. In terms of level of education, 14 (23.3%) were illiterate, 26 (43.3%) had primary education, 15 (25%) had secondary education, and 5 (8%) had a high school diploma or a higher degree.

**Table 1:** Sample characteristics: demographic variables

		Frequency	Percent
Sex	Female	30	50
	Male	30	50
Age	30-40	17	28/3
	40-50	30	50
	50-60	13	21/7
Married	Single	42	70
	Married	18	30
Inhabitancy on center	Under 2 years	1	1/7
	2-4 years	14	23/3
	4-6 years	24	40
	6-8 years	11	18/3
	8-10 years	8	13/3
	More than 10 years	2	3/3
Education level	Illiterate	14	23/3
	Primary	26	43/3
	Guidance	15	25
	Diploma and more	5	8

**Table 2:** The result of person correlation

*Variable*	*number*	*sexuality*	*correlation*	*significant*
Depression & SQLS	30	female	-0.31	0.043	(1-tailed)
	30	male	-0.32	0.041	
	60	Female & male	-0.30	0.01

**Table 3:** Coefficient among the level of SQLS & Depression

*Variable*		*female*		*Male*	
	
		correlation	significant	correlation	significant
Depression&	Motivation	-0.24	0.10	-0.36	0.02
	Symptom	-0.45	0.006	-0.12	0.26
	Psychosocial	-0.08	0.34	-0.39	0.01

**Table 4:** Predictors of SQLS — Regression Analysis by enter method

	Predictor	ß value	R^2^ value	Adjusted R^2^ value	F value	Significance	Confidence interval	
**Female**			0.51	0.180	3.12	0.043	0.756	2.44
	Motivation	0.25				**0.22**	-0.026	0.107
	Symptom	0.51				**0.01**	0/013	0.096
	Psychosocial	-0.28				**0.21**	-0.053	0/012
**Male**			0.52	0.184	3.18	0.041	-0.924	1.55
	Motivation	0.296				**0.14**	-0.017	0.12
	Symptom	-0.390				0.12	-0.075	0.01
	Psychosocial	.533				**0.04**	-0003	0.09

Pearson's correlation test was employed to determine the relationship between depression and quality of life in schizophrenic patients. In this study, P<0.05 was used as the significance level. There was a significant negative correlation between high level of depression and low quality of life (one-tailed test, P=0.01, n=60, r=-0.30). (Women: one-tailed test, P=0.043, n=30, r=-0.31; Men: one-tailed test, P=0.041, n=30, r=-0.32).

Multivariate regression analysis was employed to determine the relationship between depression and the quality of life subscales. A significant model about women and men was obtained using the Enter method. (Women: P=0.043 & F3 26= 3.12 & Adjusted R2 value=0.18; Men: P=0.041 & F3 26= 3.18 & Adjusted R2 value=0.18). This model explains 18% of the variance. In case of women, the variable of symptoms and complications was a significant predictor; however, the other two variables (energy and motivation and psychosocial) were not significant predictors. In case of men, psychosocial variable was a significant predictor; however, the other two variables (energy and motivation and symptoms and complications) were not significant predictors.

## 4. Discussion

Investigating the study hypotheses showed that there was a negative correlation between the high level of depression and low quality of life. According to Strauss et al. (2012), negative symptoms and depression are a predictive factor for well-being in schizophrenic patients. According to Naber et al. (2013), fewer symptoms of depression and higher quality of life in schizophrenic patients can predict early recovery in psychopathology, quality of life, and well-being. The studies conducted by Babinkostova et al. (2011), Kao et al. (2011), Renwick et al. (2012), and Conton et al. (2010), Priebe et al. (2011) also confirm this result. In a study titled “A comparative study on quality of life of patients of schizophrenia with and without depression”, Dan et al., (2011) showed that the overall score of depression had no relationship with the quality of life in schizophrenic patients; however, symptoms of psychopathology had a strong negative correlation with the quality of life. Konarzewska et al. (2012) showed that symptoms of depression and alcohol dependence do not affect the MAST score of schizophrenic patients; however, dissatisfaction with the quality of life had a relationship with higher MAST scores only in schizophrenic alcohol-dependent women. These different results might be due to cultural differences, implementation method, difference in sample size and demographic characteristics, difference in the type of questionnaire, and place of implementation. According to Taghavi et al. (2008), the frequency of depression in schizophrenic patients under treatment was 39.9%, which was high in females, and single, divorced, unemployed, and illiterate individuals. Moreover, the level of depression in acute and complete remission phases was high and low respectively. In his study on Chinese schizophrenic patients, Yan (2012) found that quality of life had an impact on suicidal thoughts. Sense of coherence and acceptance of disease and depression in schizophrenic patients affect the quality of life in such patients (Badura-Brzoza, 2012). Concerning the second hypothesis, the results of multivariate regression analysis in determining the relationship between depression and the quality of life subscales showed that in women, the variable of symptoms and complications was a significant predictor; however, the other two variables (energy and motivation and psychosocial) were not significant predictors. Despite new antipsychotics, such patients still suffer from poor social functioning and a high level of physical discomfort, which reduces their quality of life (Hwang, 2009). Since women pay more attention to physical symptoms and their bodies than men, symptoms and complications are predictive factors for depression in women. In case of men, psychosocial variable was a significant predictor; however, the other two variables (energy and motivation and symptoms and complications) were not significant predictors. In his studies, Renwick (2012) showed that depression had reduced the quality of life in these patients in areas such as psychological well-being and social relations, which is consistent with the results of the studies by Cotton (2010). In our study population, the men and women had no significant difference in terms of demographic variables such as level of education, marital status, age, and length of stay. There was a difference only in ethnic factor, including native and non-native (P=0.004). In women, 53.3% were native and 46.7% were non-native. In men, 86.7% were native and 13.3% were non-native. This shows that the two groups are different in terms of ethnic factor, which can justify the difference between men and women in terms of predictor variables of depression. Moreover, in our study population, men and women had no difference in terms of depression (P=0.84, df=58, n=60, t=0.2); however, they were different in terms of quality of life (P=0.024, df=58, n=60, t=2.32) and women enjoyed a higher quality of life, which might be due to men's paying more attention to psychosocial factors. A study conducted by Khodadadi et al., (2010) on the quality of life in schizophrenic patients in terms of individual, social, and clinical characteristics showed that 40.4% of these samples were really dissatisfied with having negative psychological feelings. A study by Solanki et al. (2010) also shows that the lowest quality of life score in schizophrenic patients was observed in social communication dimension. According to the World Health Organization, quality of life means individuals’ perception of their lives based on their culture, system of values, goals, expectations, standards, and concerns (Divanon et al., 2006). This perception might be different in men and women and cause difference in predictive factors for depression. In general, research has shown that symptoms of depression in schizophrenia can lead to dissatisfaction with the quality of life, impairment in psychological functioning, higher recurrence rate, longer hospitalization, lack of response to medical treatment, occupational impairment, less activity, cognitive impairment, poor social functioning, substance abuse, negative attributional style, and suicide (Hausmann et al., 2002; Siris, 2000).
